# Sorafenib Prevents Escape from Host Immunity in Liver Cirrhosis Patients with Advanced Hepatocellular Carcinoma

**DOI:** 10.1155/2012/607851

**Published:** 2012-05-14

**Authors:** Hidenari Nagai, Takanori Mukozu, Daigo Matsui, Takenori Kanekawa, Masahiro Kanayama, Noritaka Wakui, Kouichi Momiyama, Mie Shinohara, Kazunari Iida, Koji Ishii, Yoshinori Igarashi, Yasukiyo Sumino

**Affiliations:** Division of Gastroenterology and Hepatology, Toho University Medical Center, Omori Hospital, 6-11-1, Omorinishi, Ota-ku, Tokyo 143-8541, Japan

## Abstract

*Purpose*. It has been reported that Th2 cytokines downregulate antitumor immunity, while activation of type T cells promotes antitumor immunity. The aim of this paper was to evaluate host immunity in liver cirrhosis (LC) patients with advanced hepatocellular carcinoma (aHCC) receiving sorafenib therapy. *Methods*. Forty-five adult Japanese LC patients received sorafenib for aHCC between 2009 and 2011 at our hospital. Sorafenib was administered at a dose of 200–800 mg/day for 4 weeks. Blood samples were collected before and after treatment. *Results*. Eleven patients were treated with sorafenib at 200 mg/day (200 group), 27 patients received sorafenib at 400 mg/day (400 group), and 7 patients were given sorafenib at 800 mg/day (800 group). There was no significant change in the percentage of Th1 cells after treatment in any group. However, the percentages of Th2 cells and regulatory T cells were significantly decreased after treatment in the 400 group and 800 group compared with before treatment, although there was no significant change after treatment in the 200 group. *Conclusions*. These results indicate that treatment with sorafenib might induce Th1 dominance and prevent the escape of tumor cells from the host immune system in LC patients with aHCC.

## 1. Introduction

Hepatocellular carcinoma (HCC) is the fifth most common malignancy in men and the eighth most common in women, with over 500,000 new cases being diagnosed worldwide each year [1–3]. Several therapeutic modalities, including surgery, percutaneous ethanol injection (PEI), transcatheter arterial chemoembolization (TACE), and radiofrequency ablation (RFA), are used to treat small tumors. Recently, the oral multikinase inhibitor sorafenib, which shows strong in vitro activity by targeting the Raf/mitogen-activated protein kinase/extracellular signal-related kinase signaling pathway, has been used to treat advanced hepatocellular carcinoma (aHCC). In the Sorafenib HCC Assessment Randomised Protocol (SHARP) study, 602 patients (mainly Europeans) were randomized to receive sorafenib or placebo. They had an Eastern Cooperative Oncology Group performance status of 0–2 and were all in Child-Pugh class A. The sorafenib group achieved a median overall survival time of 10.7 months versus 7.9 months for the placebo group [4]. Sorafenib has also demonstrated significant clinical activity against HCC in phase II and phase III studies [5, 6], in which treatment with this agent achieved a longer median survival time and longer time to radiologic progression compared with placebo.

When treating aHCC in patients with cirrhosis of the liver, we must consider the influence of tumor-related factors, the properties of the anticancer drugs or molecular-targeting agents, and host immunity. Tumors develop various mechanisms to escape from the host immune system and to inhibit antitumor responses. Dendritic cells (DCs) are the most potent antigen-presenting cells with respect to their ability to efficiently prime both CD4-positive and CD8-positive cytotoxic T cells. It has been reported that impaired DC function might be an important factor in allowing tumors to escape from surveillance [7], and that the number of peripheral blood DCs is significantly decreased in cancer patients [8, 9]. Production of immunosuppressive factors, an increase of regulatory (Treg) cells, and downregulation of the expression of tumor antigens and major histocompatibility complex (MHC) molecules are some of the mechanisms by which tumor cells can escape from immune recognition [10, 11]. All of these mechanisms may operate in patients with HCC. Based on their cytokine production profiles, helper T cells can be divided into two distinct populations, which are known as type 1 helper T cells (Th1 cells) and type 2 helper T cells (Th2 cells). Th1 cells produce interferon-gamma (IFN-gamma) and interleukin 2 (IL-2) and play a pivotal role in cell-mediated immunity, while Th2 cells produce interleukin 4 (IL-4), interleukin 10 (IL-10), and other cytokines that are essential for the regulation of humoral immunity [12, 13]. The Th1 subset is responsible for activation of cell-mediated immunity and cytotoxic CD8^+^ T lymphocytes (CTLs), while the Th2 subset primarily assists in B cell activation [14]. The direction in which naive CD4^+^ cells differentiate depends on their first encounter with the triggering agents. The factors regulating differentiation are still not fully understood, although the cytokine environment during the differentiation of antigen-primed CD4^+^ T helper cells is thought to determine the subset that emerges [15]. IFN-gamma preferentially inhibits the proliferation of Th2 cells, while IL-4 and IL-10 are secreted by Th2 cells and suppress the secretion of IL-12, which is the critical cytokine for Th1 differentiation [16, 17]. Thus, Th1 and Th2 cells cross-regulate their own development. It has been reported that Th2 cytokines inhibit antitumor immunity [18], while the activation of Th1 responses promotes antitumor immunity [19–22]. We have previously shown that Th1 dominance is lost due to an increase of Th2 cells in HCC patients, and that carcinogenesis might be more likely to occur in patients with chronic HCV infection and an increase of Th2 cells [23]. The response of T cells to self-and nonself-antigens is controlled by a network of Treg cells. CD4^+^ cells that constitutively express CD25, the interleukin-2-receptor *α*-chain, are generally considered to be natural Treg cells and account for 5–10% of all peripheral CD4^+^ T cells in healthy animals and humans [24–26].

We previously examined the changes of host immunity and efficacy of treatment in LC patients with aHCC receiving hepatic intra-arterial chemotherapy (HAIC). We found that the percentage of Th2 cells increased in liver cirrhosis (LC) patients with aHCC as the response to HAIC decreased. This suggested that HAIC might be not useful for patients with aHCC because it induces Th2 dominant host immunity [27, 28]. However, it is not clear how sorafenib influences host immunity in LC patients with aHCC. Accordingly, the aim of the present study was to retrospectively evaluate changes of host immunity in LC patients with aHCC receiving sorafenib therapy.

## 2. Methods

### 2.1. Patients

Forty-five adult Japanese LC patients were treated for an aHCC with sorafenib between 2009 and 2011 at our hospital. Sorafenib was administered at a dose of 200–800 mg/day for 4 weeks depending on the patient's body habitus and age. Blood samples were collected in the early morning before and after treatment.

### 2.2. Analysis of CD4-Positive T Cell Subsets

**Figure 1:**
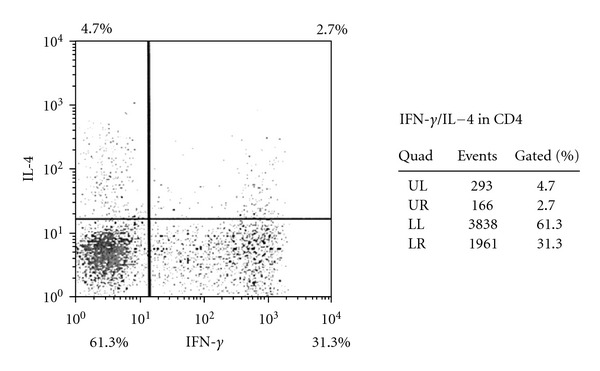
Flow cytometric detection of interferon (IFN-*γ*) and interleukin (IL-4) in CD4-positive T cells. Upper left: IFN-*γ*-negative and IL-4-positive cells (Th2); lower right: IFN-*γ*-positive and IL-4-negative cells (Th1).

**Figure 2:**
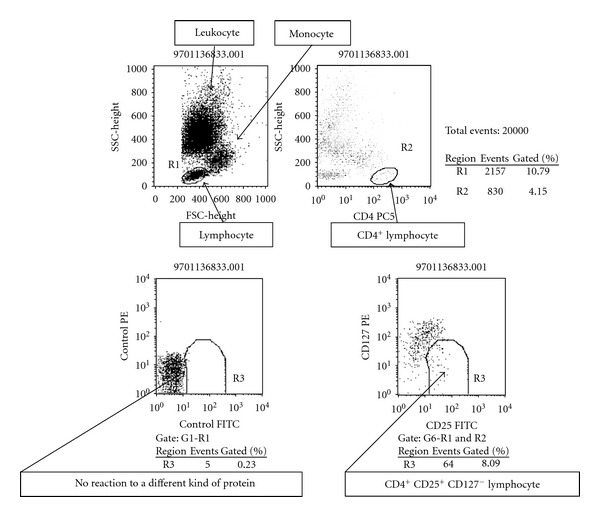
Flow cytometric detection of CD25 FITC and CD127 PE in CD4-positive T cells. Upper left: leucocytes, monocytes, and lymphocytes; Upper right: CD4-positive lymphocytes; lower left: no reaction to a different protein (control); lower right: CD4-positive and CD127-negative lymphocytes (Treg).

Peripheral blood CD4-positive T cell subsets were analyzed after nonspecific stimulation with phorbol 12-myristate 13-acetate (PMA), ionomycin, or brefeldin A (Sigma Chemical Co., St. Louis, MO, USA), according to the modified method of Jung et al. [29]. Flow cytometry was used to detect cytoplasmic expression of IFN-gamma and IL-4 by peripheral blood CD4-positive T cells after culture and staining, as reported previously. Results were expressed as the percentage of cytokine-producing cells in the CD4-positive T cell population, which was divided into IFN-gamma-positive/IL-4-negative (Th1) cells and IFN-gamma-negative/IL-4-positive (Th2) cells (Figure 1). Regulatory T cells (Treg cells) were identified as CD25^high^/CD127^low^ cells (Figure 2).

### 2.3. Evaluation of Tumor Response

Tumor responses were assessed according to the modified Response Evaluation Criteria in Solid Tumors (RECIST) [30, 31].

### 2.4. Statistical Analysis

Statistical analysis was performed by using the Statistical Package for the Social Sciences (SPSS version 11.0; SPSS, Chicago, IL, USA). Results are expressed as the mean ± standard deviation (SD). Wilcoxon's signed rank sum test was used to compare patient characteristics within each group. A probability of less than 0.05 was considered to indicate statistical significance in all analyses.

## 3. Results

The 45 patients were divided into three groups. Eleven patients were administered sorafenib at a dose of 200 mg/day for 4 weeks (200 group), 27 patients were administered 400 mg/day for 4 weeks (400 group), and 7 patients were administered 800 mg/day for 4 weeks (800 group). There were 7 men and 4 women aged 60 to 82 years (mean ± SD: 72.1 ± 7 years) in the 200 group, 24 men and 3 women aged 56 to 79 years (mean ± SD: 69.4 ± 6 years) in the 400 group, and 7 men aged 61 to 80 years (mean ± SD: 66.1 ± 7 years) in the 800 group. In the 200 group, eight patients had HCV-related LC (C-LC), one patient had HBV-related LC (B-LC), and two patients had non-B non-C LC (non-B non-LC), which did not include LC due to autoimmune diseases such as autoimmune hepatitis or primary biliary cirrhosis. In the 400 group, there were 17 patients with C-LC, 5 patients with B-LC, and 5 patients with non B non-C LC. In the 800 group, 1 patient had C-LC, 2 patients had B-LC, and 4 patients had non B non-C LC. The Child-Pugh class was A for 8 patients in the 200 group, 26 patients in the 400 group, and 5 patients in the 800 group, while it was B for 3, 1, and 2 patients, respectively. Nine patients had stage IVA disease and two patients had stage IVB disease in the 200 group. There was 1 patient with stage III disease, 24 patients with stage IVA disease, and 2 patients with stage IVB disease in the 400 group, while all 7 patients had stage IVA disease in the 800 group. Eight patients had a Japan Integrated Staging (JIS) score [32] of 3, and three patients had a score of 4 in the 200 group, while the respective numbers were 26 and 1 in the 400 group, as well as 5 and 2 in the 800 group (Table 1). In the 200 group, one patient had involvement of the major branches of the portal vein, and there were no patients with portal trunk thrombus, while the respective numbers were 3 and 4 in the 400 group, as well as 1 and 1 in the 800 group. In the 800 group, one patient had invasion of the main hepatic venous trunk.

### 3.1. Response

Table 2 summarizes the response to treatment. In the 200 group, 8 of the 11 patients (72.7%) showed progressive disease (PD) and 2 patients (18.2%) had stable disease (SD), but no patient achieved a partial response (PR). In the 400 group, 4 of the 27 patients (14.8%) achieved PR, while 9 patients (33.3%) showed PD and 11 patients (40.7%) had SD. In the 800 group, 1 of the 7 patients (14.3%) achieved PR, while 3 patients (42.9%) patients showed PD and 3 patients (42.9%) had SD.

### 3.2. Peripheral Blood Th1 and Th2 Cells

**Figure 3:**
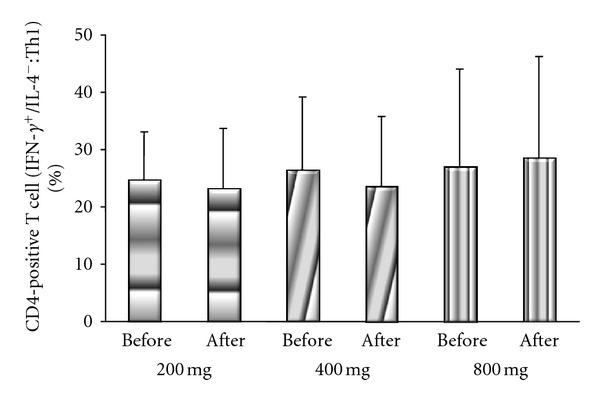
Comparison of the IFN-*γ*-positive and IL-4-negative (Th1) subset of CD4-positive T cells before and after treatment in the 200 group, 400 group, and 800 group. There were no significant differences between before and after treatment in any group.

**Figure 4:**
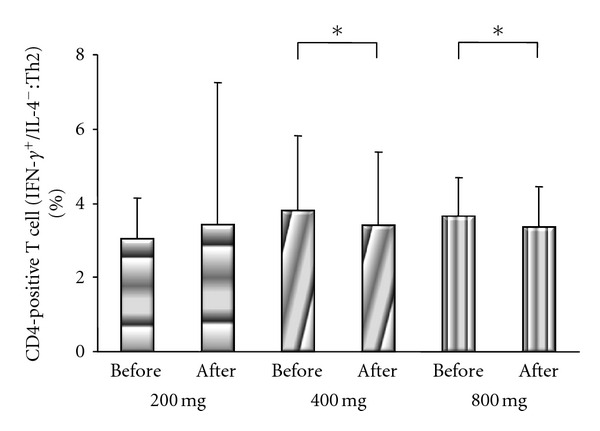
Comparison of the IFN-*γ* negative and IL-4 positive (Th2) subset of CD4-positive T cells before and after treatment in the 200 group, 400 group, and 800 group. There were significant differences of Th2 cells between before and after treatment in the 400 group and 800 groups (*P* < 0.05 by Wilcoxon's signed rank sum test), but there was no significant difference of 14 Th2 cells in the 200 group.

There were no significant differences of Th1 cells between before treatment (200 group: 26.3 ± 8%; 400 group: 27.6 ± 11%; 800 group: 27.7 ± 17%) and after treatment (200 group: 23.8 ± 10%; 400 group: 24.9 ± 11%; 800 group: 28.7 ± 18%) in each of the 3 groups (Figure 3). In contrast, significant differences of Th2 cells were noted in the 400 and 800 groups between before treatment (400 group: 3.9 ± 2%; 800 group: 3.7 ± 1%) and after treatment (400 group: 3.5 ± 2%; 800 group: 3.3 ± 1%) (*P* = 0.014 and *P* = 0.028, respectively, by Wilcoxon's signed rank sum test), although there was also no significant difference of Th2 cells between before and after treatment (3.1 ± 1% versus 3.3 ± 3%) in the 200 group (Figure 4).

### 3.3. Peripheral Blood Treg Cells

**Figure 5:**
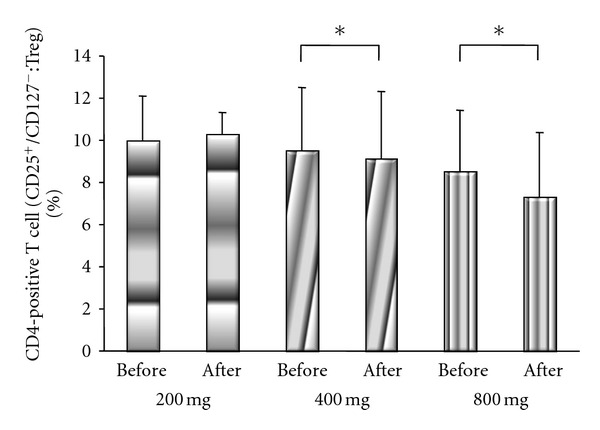
Comparison of CD25 FITC and CD127 PE among CD4-positive T cells (Treg cells) before and after treatment. There were significant differences of Treg cells between before treatment and after treatment in the 400 group and 800 groups (*P* < 0.05 by Wilcoxon's signed rank sum test), but there was no significant difference of Th2 cells in the 200 group.

There were significant differences of Treg cells in the 400 and 800 groups between before treatment (400 group: 9.5 ± 3%; 800 group: 8.5 ± 3%) and after treatment (400 group: 9.2 ± 3%; 800 group: 7.3 ± 3%) (*P* = 0.026 and *P* = 0.028, respectively, by Wilcoxon's signed rank sum test), but there was also no significant difference of Th2 cells between before and after treatment (10.0 ± 2% versus 10.1 ± 3%) in the 200 group (Figure 5).

### 3.4. Host Immunity and Objective Response

**Figure 6:**
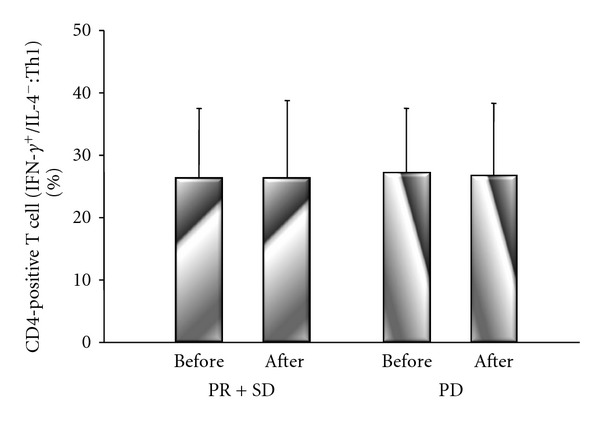
Comparison of the IFN-*γ*-positive and IL-4-negative (Th1) subset of CD4-positive T cells before and after treatment in the PR + SD group and PD group. There were no significant differences of Th1 cells between before treatment and after treatment in either group.

**Figure 7:**
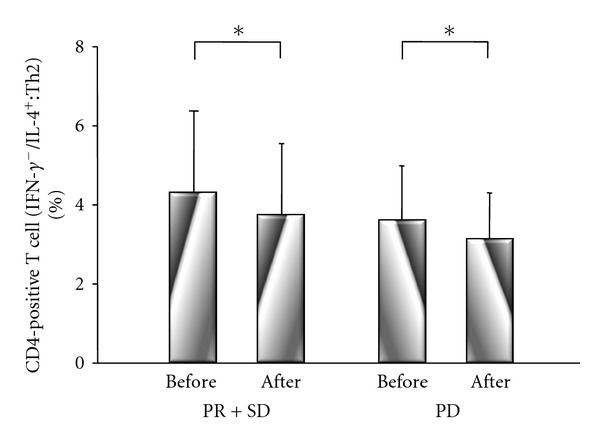
Comparison of the IFN-*γ*-negative and IL-4-positive (Th2) subset of CD4-positive T cells before and after treatment in the PR + SD group and PD group. There were significant differences of Th2 cells in the PR + SD and PD groups between before treatment and after treatment (PR + SD group: *P* = 0.017, PD group: *P* = 0.020 by Wilcoxon's signed rank sum test).

**Figure 8:**
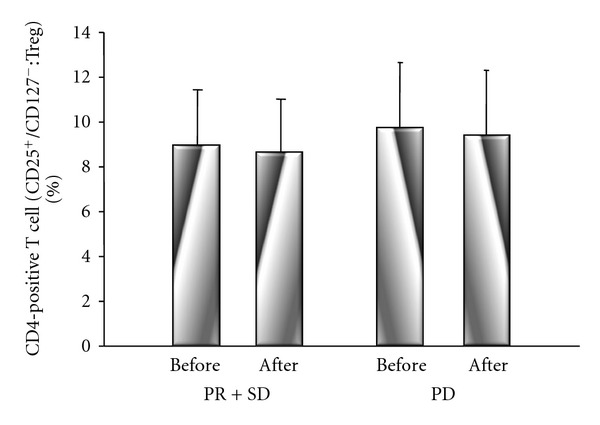
Comparison of CD25 FITC and CD127 PE among CD4-positive T cells (Treg cells) before and after chemotherapy. There were no significant differences of Treg cells between before treatment and after treatment in either group, although Treg cells decreased after treatment in both groups.

There were no significant differences of Th1 cells between before treatment (PR + SD group: 26.2 ± 11%; PD group: 26.8 + 10%) and after treatment (PR + SD group: 26.2 ± 12%; PD group: 26.3 ± 12%) in either group (Figure 6). However, there were significant differences of Th2 cells in the PR + SD and PD groups between before treatment (PR + SD group: 4.2 ± 2%; PD group: 3.6 ± 1%) and after treatment (PR + SD group: 3.7 ± 2%, *P* = 0.017; PD group: 3.1 ± 1%, *P* = 0.020) (Figure 7). There were no significant differences of Treg cells between before treatment (PR + SD group: 8.9 ± 2%; PD group: 9.6 ± 3%) and after treatment (PR ± SD group: 8.6 ± 2%; PD group: 9.3 ± 3%) in either group, although Treg cells decreased after treatment in both groups 14 (Figure 8).

## 4. Discussion

The oral multikinase inhibitor sorafenib has revolutionized the treatment of aHCC in patients with LC. It has been reported that sorafenib therapy prolongs the median overall survival of patients with aHCC [4], but there have been few reports about the influence of sorafenib on host immunity in a HCC patients. Kohga et al. demonstrated that a disintegrin and metalloproteinase 9 (ADAM9) were overexpressed in human HCC tissues, while ADAM9 knockdown increased the expression of membrane-bound MHC class I-related chain A (MICA), decreased the production of soluble MICA, and increased the sensitivity of human HCC cells to natural killer (NK) cells. Furthermore, they indicated that sorafenib enhanced the sensitivity of HCC to NK cells via inhibition of ADAM9 protease activity and modification of MICA expression [33]. However, it has been unclear whether sorafenib reverses tumor escape mechanisms from host immunity after recognition of MICA expression. Zhao et al. demonstrated that sorafenib inhibited the proliferation of T cells and induced T cell apoptosis and they suggested that sorafenib may impair T cell-related immunity by inducing apoptosis [34]. In addition, Madeleine et al. reported that sorafenib significantly reduced the induction of antigen-specific T cells, impaired the intracellular signaling cascades in DCs, and induced apoptosis of DCs. They concluded that sorafenib interferes with the function and maturation of monocyte-derived DCs [35]. However, it has been unclear whether sorafenib causes similar changes in LC patients with aHCC. The present study showed that there were no significant changes of Th1 cells after treatment in each of the 3 treatment groups. In contrast, the percentage of Th2 cells showed a significant decrease after treatment in the 400 and 800 groups, although there was no significant difference in the 200 group. These results indicate that treatment with sorafenib at doses of 400 mg/day or more can shift host immunity from Th2 dominance to Th1 dominance in LC patients with aHCC, although sorafenib does not increase number of Th1 cells.

There are two distinct subsets of Treg cells in the peripheral lymphoid organs, which are natural Treg (nTreg) cells that develop in the thymus after recognition of high-affinity autoantigens, and induced Treg (iTreg) cells that develop from conventional T cells after peripheral exposure to antigens and cytokines such as TGF-*β* or IL-10 [36]. These subsets of the Treg network may have a synergistic action or may have different targets that maintain immune homeostasis, although they possibly even have a developmental role [37]. An increase of circulating and tumor-infiltrating FoxP3+ Treg cells has been reported in HCC patients [38]. Sorafenib is the first systemic agent approved for treating HCC and is a multikinase inhibitor with activity against VEGFR2, PDGFR, c-Kit receptor, b-RAF, and p38 [39], which are signal transduction pathways that may be involved in the pathogenesis of HCC [40]. Sorafenib simultaneously inhibits several components of the Raf-MEK-ERK signaling pathway, thus preventing tumor growth and VEGFR-1, VEGFR-2, VEGFR-3, and PDGFR-b, to inhibit neoangiogenesis [41]. In the present study, the percentage of Treg cells in the 400 group and the 800 group showed a significant decrease after treatment compared with before treatment, although there was no significant difference after treatment in the 200 group. These results indicate that sorafenib therapy at doses ≥400 mg/day inhibited Treg cells and induced Th1 dominant host immunity in our LC patients with aHCC. It is possible that sorafenib achieved this by decreasing iTreg cells through a reduction of nTreg exposure to HCC antigens by inhibiting tumor neoagiogenesis.

In the present study, the percentage of Th2 cells showed a significant decrease after treatment in both the PR + SD group and the PD group, although there was no significant change of Th1 cells after treatment in either group. In contrast, there were no significant differences of Treg cells between before and after treatment in either group, although these cells decreased after treatment in both groups. These results indicate that treatment-related changes of host immunity in LC patients with aHCC might not influence the objective response to sorafenib.

**Figure 9:**
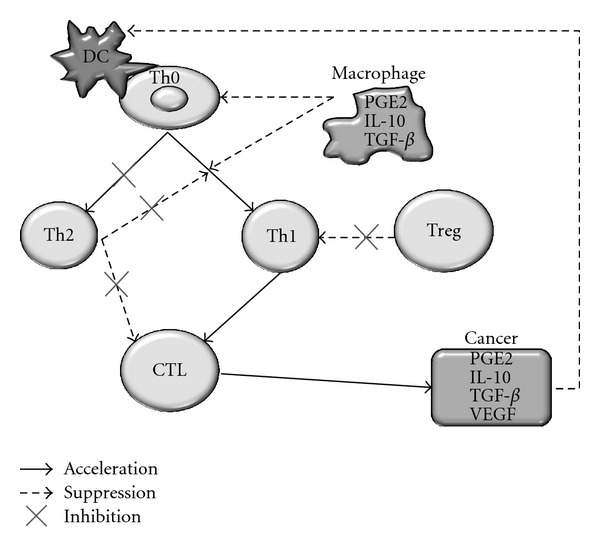
Possible effect of sorafenib on host immunity. Sorafenib therapy might abrogate escape mechanisms from the host immunity in LC patients with aHCC by inducing Th1 dominance. DC: Dendritic cell, Treg: regulatory T cells, Th1: type 1 helper T cells, Th2: type 2 helper T cells, CTL: cytotoxic CD8^+^ T lymphocytes.

In conclusion, we demonstrated that administration of sorafenib at doses >400 mg/day induced Th1 dominant host immunity in LC patients with aHCC. This effect of sorafenib therapy might be dependent on two mechanisms, which are (1) induction of antigen-primed CD4^+^ T helper cells after recognition of MICA expression by HCC cells and (2) a decrease of Treg cells related to inhibition of tumor neoangiogenesis. It is also possible that sorafenib might induce T cell apoptosis or interfere with the function and maturation of monocyte-derived DCs. Sorafenib therapy at doses >400 mg/day has the potential to abrogate the mechanisms of tumor escape from the host immune system in LC patients with aHCC by inducing Th1 dominance (Figure 9). Accordingly, neoadjuvant therapy with sorafenib before induction of chemotherapy might prolong the survival or improve the objective response of LC patients with aHCC receiving HAIC by modifying host immunity.

## Figures and Tables

**Table 1:** Clinical characteristics of 45 liver cirrhosis patients with HCC.

Dose of sorafenib	200 mg	400 mg	800 mg
No. of patients	11	27	7
Mean age	72.1 ± 7	69.4 ± 6	66.1 ± 7
Gender (M/F)	7/4	24/3	7/0
Type of cirrhosis (HBV/HCV/non B non C)	1/8/2	5/17/5	2/1/4
Child-Pugh classification (A/B/C)	8/3/0	26/1/0	5/2/0
Stage (III/IVAIIVB)	0/9/2	1/24/2	0/7/0
JIS score (2/3/4/5)	0/8/3/0	0/26/1/0	0/5/2/0

**Table 2:** Objective responses of liver cirrhosis patients with advanced HCC treated after 4–8 weeks of sorafenib treatment.

Dose of sorafenib	PR	SO	PO	Response rate (%)
200 m g (*n* = 11) (1 dropout)	0	2	8	0.0
400 mg (*n* = 27) (3 dropout)	4	11	9	16.7
800 mg (*n* = 7) (0 dropout)	1	3	3	14.3
